# A Comprehensive Analysis of the Lysine Acetylome in the Aquatic Animals Pathogenic Bacterium *Vibrio mimicus*

**DOI:** 10.3389/fmicb.2022.816968

**Published:** 2022-02-17

**Authors:** Junlin Wang, Huanying Pang, Linlin Yin, Fuyuan Zeng, Na Wang, Rowena Hoare, Sean J. Monaghan, Wanxin Li, Jichang Jian

**Affiliations:** ^1^Fisheries College of Guangdong Ocean University and Southern Marine Science and Engineering Guangdong Laboratory (Zhanjiang), Zhanjiang, China; ^2^Guangdong Provincial Key Laboratory of Pathogenic Biology and Epidemiology for Aquatic Economic Animals and Key Laboratory of Control for Diseases of Aquatic Economic Animals of Guangdong Higher Education Institutes, Zhanjiang, China; ^3^Chinese Academy of Inspection and Quarantine, Beijing, China; ^4^Institute of Aquaculture, University of Stirling, Stirling, United Kingdom; ^5^School of Public Health, Fujian Medical University, Fuzhou, China

**Keywords:** *Vibrio mimicus*, pathogen, lysine acetylation, acetylome, virulence

## Abstract

Protein lysine acetylation is an evolutionarily conserved post-translational modification (PTM), which is dynamic and reversible, playing a crucial regulatory role in almost every aspect of metabolism, of both eukaryotes and prokaryotes. Several global lysine acetylome studies have been carried out in various bacteria, but thus far, there have been no reports of lysine acetylation for the commercially important aquatic animal pathogen *Vibrio mimicus.* In the present study, we used anti-Ac-K antibody beads to highly sensitive immune-affinity purification and combined high-resolution LC-MS/MS to perform the first global lysine acetylome analysis in *V. mimicus*, leading to the identification of 1,097 lysine-acetylated sites on 582 proteins, and more than half (58.4%) of the acetylated proteins had only one site. The analysis of acetylated modified peptide motifs revealed six significantly enriched motifs, namely, KacL, KacR, L(-2) KacL, LKacK, L(-7) EKac, and IEKac. In addition, bioinformatic assessments state clearly that acetylated proteins have a hand in many important biological processes in *V. mimicus*, such as purine metabolism, ribosome, pyruvate metabolism, glycolysis/gluconeogenesis, the TCA cycle, and so on. Moreover, 13 acetylated proteins were related to the virulence of *V. mimicus*. To sum up, this is a comprehensive analysis whole situation protein lysine acetylome in *V. mimicus* and provides an important foundation for in-depth study of the biological function of lysine acetylation in *V. mimicus*.

## Introduction

Post-translational modification (PTM) of proteins is a significant regulatory mechanism that affects processes beyond gene expression in bacteria. Proteins are the basis for functional life activities. The diversity of protein functions is facilitated by PTM that changes the structure/function relationship and affects the formation of protein complexes, enzyme catalysis, and other biomolecular interactions (Cain et al., 2014). In recent years, accumulating evidence indicates that PTMs play a critical role in various physiological processes, such as immunometabolism (Diskin et al., 2020), protein synthesis and turnover (Rioseras et al., 2018), protein stability (Ishigaki et al., 2017; Sang et al., 2017), dormancy (Yang et al., 2018), nitrogen metabolism (Macek et al., 2019), and virulence (Xie et al., 2019). Among the hundreds of diverse PTMs, protein phosphorylation, acetylation, succinylation, pupylation and ubiquitin-like modifications, glycosylation, and lipidation play a major role in regulating bacterial cell cycle, cell metabolism, persistence, and virulence (Ren et al., 2016, 2019; Macek et al., 2019). With the fast development of proteomics technology based on high-throughput mass spectrometry, a variety of PTMs on lysine residues have been enriched and identified (Zhang et al., 2013; Yang et al., 2015; Alpha-Bazin et al., 2020; Lei et al., 2021). Among them, lysine acetylation (K_*ac*_) is an evolutionarily conserved PTM that can be reversed by deacetylases found in prokaryotes and eukaryotes. The transfer of acetyl group to the side chain of lysine is realized by enzymatic or non-enzymatic, and the enzymatic reaction is realized with acetyl phosphate or acetyl coenzyme a as the cofactor of lysine acetyltransferase (KAT) (Carabetta and Cristea, 2017; Macek et al., 2019). At present, more and more studies on lysine acylated proteins have been reported in various bacterial pathogens, such as *Porphyromonas gingivalis* (Butler et al., 2015), *Pseudomonas aeruginosa* (Ouidir et al., 2015), *Aeromonas hydrophila* (Sun et al., 2019), *Vibrio cholerae* (Carsten et al., 2017), *Vibrio vulnificus* (Pang et al., 2020), and so on. In addition, acetylation modification occurs on enzymes in several central metabolic processes of bacteria, such as tricarboxylic acid cycle, glycolysis/gluconeogenesis, and pyruvate metabolism (Li W.X. et al., 2020).

*Vibrio mimicus* (*V. mimicus*) is a Gram-negative bacterium which can infect Koi carp (*Cyprinus carpio*) (Cen et al., 2013), yellow catfish (*Pelteobagrus fulvidraco*) (Geng et al., 2014; Fu et al., 2021), shrimp (Iliana et al., 2016; Boonchuen et al., 2021), and oyster (Boonchuen et al., 2021), causing severe economic losses to the aquaculture industry and threatening food safety. In addition to being a pathogen of aquatic animals, human consumption of fishery products infected by *V. mimicus* will cause symptoms such as diarrhea, nausea, vomiting, abdominal spasm, and fever (Shandera et al., 1983; Kay et al., 2012). This bacterium is closely related to *Vibrio cholerae* and has similar morphology and growth characteristics. Some researchers have reported that *V. mimicus* can produce a variety of virulence factors such as adhesins, hemolysins, and several proteases (collagenases and metalloproteases), siderophores, cytolysins, lipases, and DNAses (Baffone et al., 2001; Singh et al., 2002; Hernández-Robles et al., 2021), so as to make microorganisms invade the host and cause tissue damage, in order to obtain the nutrient source required for its growth and reproduction (Singh et al., 2002; Hernández-Robles et al., 2021). However, the pathogenic mechanisms of *V. mimicus* are largely unknown, so it is difficult to prevent it during production.

As the outbreak of vibriosis becomes more serious and frequent, there is an urgent need to strengthen the study of acetylation modification on the regulation of pathogenic mechanisms. More and more reports show that the study of acetylation modification in bacterial pathogens has important functions. However, so far, no acetylated protein in *V. mimicus* has been reported, which is a major obstacle to grasp the regulatory mechanism of Kac in this pathogen. Therefore, we conducted a comprehensive and systematic analysis to determine the role of acetylation modification in *V. mimicus*. A total of 582 proteins and 1,097 modification sites were identified by mass spectrometry. VFDB (Virulence Factor Database) analysis revealed that 13 acetylated proteins were involved in the regulation of bacterial virulence. The results of this study show that protein acetylation modification has significant biological functions in *V. mimicus*, which lays the foundation for further research on the functional role of protein acetylation modification in bacterial virulence and other cellular processes.

## Materials and Methods

### Bacterial Strain and Protein Extraction

The *Vibrio mimicus* strain was isolated from the hepatopancreas of diseased shrimp, *Litopenaeus vannamei*, in Guangdong Ocean University, Guangdong province, China. This strain was grown in TCBS (thiosulfate citrate bile salts sucrose) agar culture medium, and the optimum culture temperature was 28°C. Protein extraction was as follows: the strain was grown in DMEM (Dulbecco’s modified Eagle medium) medium overnight (16–18 h) and transferred to fresh DMEM medium at 1% the next day. When OD_600n*m*_ was about 1.0, bacterial precipitation was collected by centrifugation at 4°C and 10,000 rpm for 5 min and washed twice with precooled PBS (pH = 7.4) buffer. The pellet was dissolved in 8 M urea, 0.2% SDS, and protease inhibitor in 50 mM Tris-HCl (pH = 8) and mechanically disrupted by ultrasonic on ice for 15 min with 9 s intervals. The broken cell solution was centrifuged at 12,000 g at 4°C for 15 min to separate the protein in the supernatant.

### Trypsin Digested Proteins and Enrichment of Lysine-Acetylated Peptides by Anti-acetyl-lysine Beads

Protein (20 mg) was reduced with 10 mM DTT (dithiothreitol) at 37°C for 2 h. After cooling, 50 mM IAA (iodoacetamide) was added for alkylation at room temperature in the dark for 30 min. Add five times the volume of ddH_2_O, dilute the urea concentration to 1.5 M, add Trypsin Gold (Promega) at a ratio of 20:1, and digest at 37°C for 16–18 h. After digestion, SPE C18 column (Waters Inc., Milford, MA, United States) was used to peptide desalted and then lyophilized with centrivap vacuum concentrator (Labconco Inc., Kansas City, MO, United States). The lysine-acetylated peptides were enriched by immunoaffinity using anti-Ac-K antibody beads [PTMScan Acetyl-Lysine Motif (Ac-K) Kit, Cell Signal Technology], as previously described (Bouchut et al., 2015). Briefly, the peptides were mixed with anti-acetyl-lysine beads (Cell Signaling Technology) for 2.5 h at 4°C in MOPS IAP buffer (50 mM MOPS, 10 mM KH_2_PO_4_, and 50 mM NaCl in 1MTris, pH = 7.0) and then centrifuged for 30 s at 3,000 g at 4°C. Finally, the peptides were eluted with 0.15% TFA. The peptides were desalted using a peptide desalting rotating column (Thermo Fisher Scientific, Germany) before MS identification.

### LC-MS/MS Analysis

An EASY-nLC™ 1200 UHPLC system (Thermo Fisher Scientific, Germany) coupled to an Orbitrap Q Exactive HF-X mass spectrometer (Thermo Fisher Scientific, Germany) was used to perform Shotgun proteomics analyses in the data-dependent acquisition (DDA) mode. The specific details have been described previously (Pang et al., 2019).

### Data Analysis and Peptide Identification

The resulting MS/MS data were processed using Proteome Discoverer 2.2 software and searched according to UniProt *V. mimicus* (3,762 proteins) database. Search parameters are set as follows: trypsin was set as cleavage enzyme, a maximum of two missing cleavages are allowed, fixed modification was carbamidomethyl, and variable modifications were methionine oxidation and lysine acetylation. The length of peptide is at least 7 amino acids, and the total FDR (false discovery rate) of protein, peptide, and acetylated site was < 5% (Liu et al., 2018).

### Bioinformatic Analysis

Gene Ontology (GO) and Kyoto Encyclopedia of Genes and Genomes (KEGG) classification and enrichment analysis were performed in online omicsbean software^1^ and combined with Graphpad prism 8.0 software to form visual graphics. Amino acid sequence motifs were analyzed by online software MoMo, and *p*-value threshold was set as 10^––6^ (Modification Motifs, version 5.1.1)^2^ (Alice et al., 2018). The protein--protein interaction (PPI) network is going to receive from the STRING database,^3^ and results are displayed with Cytoscape software. The MS proteomics data have been stored to the ProteomeXchange Consortium^4^ *via* the iProX (integrated proteome resources) cooperation partner repository with the dataset identifier PXD028467.

### Co-immunoprecipitation and Western Blot Verification

The target proteins were precipitated with specific polyclonal antibodies against LuxO and LuxR. *V. mimicus* strain whole protein (500 μg) was incubated with LuxO and LuxR antibody at 4°C overnight, as previously described (Pang et al., 2020). Briefly, the protein A/G beads were washed three times with PBS buffer and then added to the lysate for incubation at 4°C for 1–3 h. The beads were centrifuged at 4°C and 3,000 g for 5 min to remove the supernatant, and washed five times with PBS buffer. In total, 50 μl loading buffer [containing 250 mM Tric-HCl pH = 6.8, 10% SDS (sodium dodecyl sulfate), 0.5% BPB (bromophenol blue), 50% glycerol, and 5% β-mercaptoethanol] was added and boiled for 5 min, and then SDS-PAGE and Western blot verification was run.

For Western blotting verification, protein was run on 10% SDS-PAGE and transferred to a PVDF (polyvinylidene fluoride, Millipore, Billerica, MA, United States) membrane by using a Trans-Blot Turbo Transfer System (Bio-Rad, Hercules, CA, United States) at 1.3 mA for 30 min. The membrane was blocked in QuickBlock™ blocking buffer for Western blot (Beyotime Biotechnology, Shanghai, China) and incubated for 5–15 min at room temperature, then probed with the anti-LuxO (1:4,000), anti-LuxR (1:4,000), and anti-acetyllysine mouse mAb (PTM-101, 1:5,000, PTM Biolabs Inc., Hangzhou, China), and incubated at 4°C for overnight. Secondary antibody is horseradish peroxidase (HRP) goat anti-mouse IgG (H + L) at a 1:8,000 dilution in QuickBlock™ secondary antibody dilution buffer (Beyotime Biotechnology, Shanghai, China). After incubation for 2 h at room temperature, the membrane was washed with TBST (20 mM Tris, 137 mM NaCl, 0.1% Tween-20, pH = 7.6) four times, visualized using Clarity™ Western ECL Substrate (Bio-Rad, Hercules, CA, United States), and recorded with Tanon-5200 Chemiluminescent Imaging System (Tanon Science &Technology Co., Ltd., Shanghai, China). Western blot experiment was repeated at least three times.

## Results and Discussion

### Comprehensive Analysis of Lysine Acetylated Proteins in *Vibrio mimicus*

**FIGURE 1:**
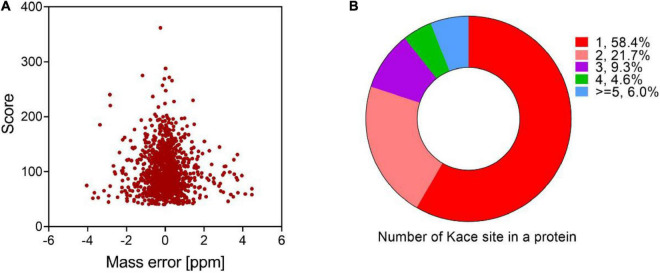
Proteome-wide identification of acetylated peptides in *V. mimicus*. **(A)** The volcano plot shows the peptide mass error (ppm) distribution of *m/z* of acetylated peptides identified. **(B)** The pie chart shows the proportion of different acetylation sites in the acetylated proteins.

The protein acetylome of *V. minicus* was determined based on antibody immunoaffinity purification and HR (high-resolution) LC-MS/MS. The mass error distribution of all identified peptides was observed by volcanic map. The results showed that most of them ranged from -2 to 2 ppm, indicating that MS data had a high mass quality accuracy (Figure 1A). In total, 1,097 acetylation sites in 582 proteins were identified, indicating that about 15.5% of the proteins in *V. mimicus* were acetylated (Supplementary Tables 1, 2). Compared with the bacterial acetylomes of other fish pathogens studied, the proportion of acetylation proteins in this bacterium is lower than *Vibrio alginolyticus* (27.1%) and *Aeromonas hydrophila* (21.7%) (Pang et al., 2019; Sun et al., 2019), but is higher than *Vibrio parahemolyticus* (13.6%) (Pan et al., 2014).

We evaluated the distribution of lysine acetylated sites and showed that most proteins were single acetylation sites (58.4%), and more than five acetylated sites accounted for 6% (Figure 1B). DnaK, RpoB, ValS, InfB, and RpoC are binding proteins with 10 or more acetylated sites, especially chaperone protein DnaK has 14 modification sites. Previous studies have shown that chaperone protein DnaK is essential for survival and contributes to disease pathogenesis by playing a role in evasion of host immune responses (Singh et al., 2007; Fourie and Wilson, 2020), suggesting that acetylated DnaK protein may play a considerable regulatory role in biological processes, such as adhesion, immune system, and heat, oxidative, and antibiotic stress. Supplementary Table 1 shows detailed information on the identified acetylated proteins and sites. The length of most peptides ranged between 7 and 23 amino acids (Supplementary Figure 1).

### Kac Motifs and Structural Properties

**FIGURE 2:**
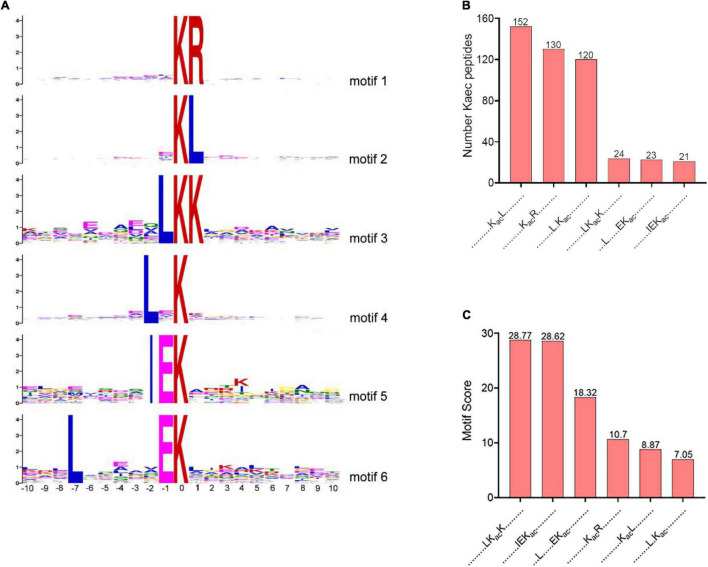
Kac motifs through MoMo software. **(A)** Acetylation sequence motifs for 10 upstream and 10 downstream amino acid sequences around the Kac. **(B)** The number of acetylated peptides determined in each conserved motif. **(C)** Motif score in each conserved motif.

To determine the lysine acetylation preferences in *V. minicus*, MoMo software was used to analyze the motifs of the 10 upstream and 10 downstream amino acid sequences of Kac in all the identified acetylated peptides. Six conserved lysine acetylated motifs were identified (Figure 2A). K_*ac*_L, K_*ac*_R, and L.K_*ac*_ (“K_*ac*_” for acetyl-lysine, and “.” represents a random amino acid residue) conserved motif accounted for 13.86, 11.85, and 10.94% of the total acetylated peptides, respectively (Figure 2B). Figure 2C provides an illustration sorting the motif scores from high to low. The motif analysis revealed that the highest enrichment of leucine (L) and arginine (R) was observed in the -2 to + 2 positions. Motif K_*ac*_R has been reported in other species of bacteria and plants, such as *Streptococcus pneumoniae*, *Vibrio cholerae*, *Cyanobacterium Synechocystis* sp., wheat (*Triticum aestivum* L.), and *Zea mays* (Mo et al., 2015; Carsten et al., 2017; Liu et al., 2018; Guo et al., 2020; Yan et al., 2020), indicating that the K_*ac*_R motif is conserved and widespread. Motif K_*ac*_L and L.K_*ac*_ have been reported in *Brenneria nigrifluens* (Li Y. et al., 2020). The other motifs, LK_*ac*_K, L(-7) EK_*ac*_K, and IEK_*ac*_, are rarely identified in other bacteria.

### Gene Ontology and Kyoto Encyclopedia of Genes and Genomes Annotation and Enrichment of Lysine Acetylome in *Vibrio mimicus*

**FIGURE 3:**
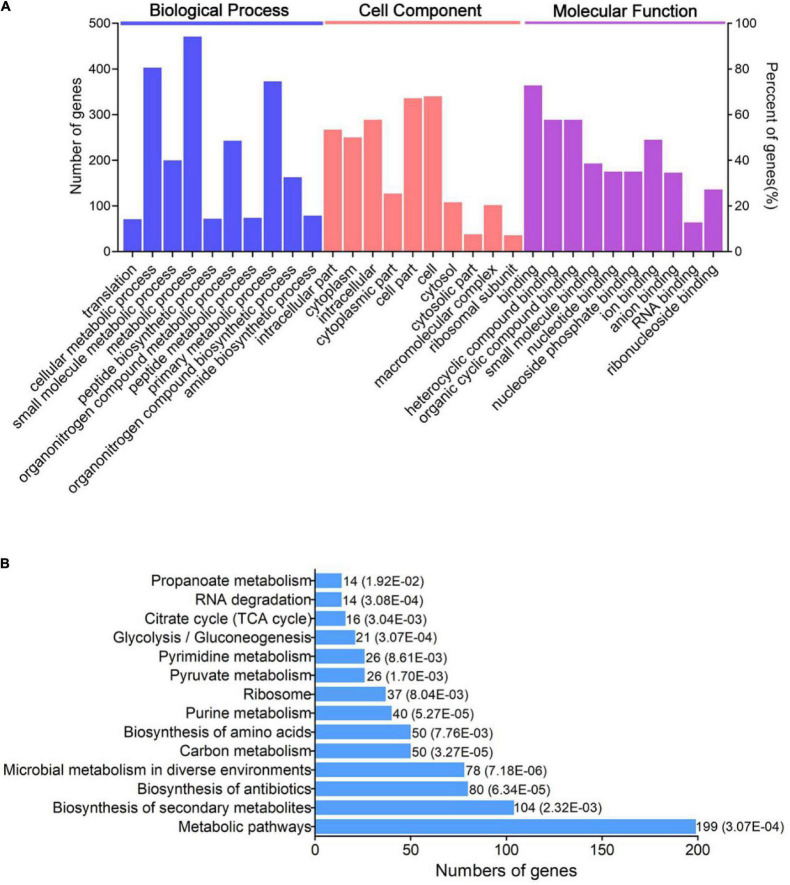
Bioinformatics analysis of acetylated proteins in *V. mimicus*. **(A)** GO-based enrichment analysis and **(B)** KEGG pathway enrichment analysis of the identified acetylated proteins.

In order to research the functional role of the lysine acetylome in *V. mimicus*, GO enrichment and KEGG pathways of acetylated proteins were performed (Figure 3). GO analysis cleared that the acetylated proteins were related to various sorts and varieties of roles in categories BP (biological process), CC (cell component), and MF (molecular function). The GO enrichment of BP declared that most of the acetylated proteins participated in metabolic activity, especially of cellular, primary, organonitrogen compound, and small molecule metabolic processes. GO categories: Cell, cell part, intracellular, intracellular part, and cytoplasm were significantly enriched in GO analysis of cell component. MF analysis revealed that acetylated proteins were outstandingly enriched in binding, heterocyclic compound binding, organic cyclic compound binding, and ion binding (Figure 3A). Consistent with previous research, the identified acetylproteins mainly enrich for metabolism processes, location of the cell, and binding function, which are especially similar to *Vibrio* spp., such as *Vibrio cholerae* V52, *Vibrio alginolyticus*, *Vibrio parahemolyticus*, and *Vibrio vulnificus* Vv180806 (Pan et al., 2014; Carsten et al., 2017; Pang et al., 2019; Pang et al., 2020).

Moreover, to further understand how the acetylated proteins participate in pathways of *V. mimicus*, cluster analysis based on KEGG pathway enrichment was conducted. Acetylated proteins were mainly concentrated in metabolic pathways, biosynthesis of secondary metabolites, biosynthesis of antibiotics, microbial metabolism in diverse environments, carbon metabolism, and so on (Figure 3B). In addition, most of the enzymes involved in energy metabolism, such as purine metabolism, pyruvate metabolism, glycolysis/gluconeogenesis, and TCA cycle, in this pathogen are acetylated. This has also been discovered in diverse organisms, which may make clear that acetylation plays a jointly regulatory role in central metabolic enzymes (Choudhary et al., 2014). Furthermore, previous research reported that large-scale ribosome-related proteins were acetylated, such as in *A. hydrophila*, *V. vulnificus*, and *S. pneumoniae*; our results also indicated the proteins involved in ribosome were more abundant through KEGG enrichment analysis (Liu et al., 2018; Sun et al., 2019; Pang et al., 2020).

### Analysis of Protein–Protein Interaction Network of Acetylated Proteins

**FIGURE 4:**
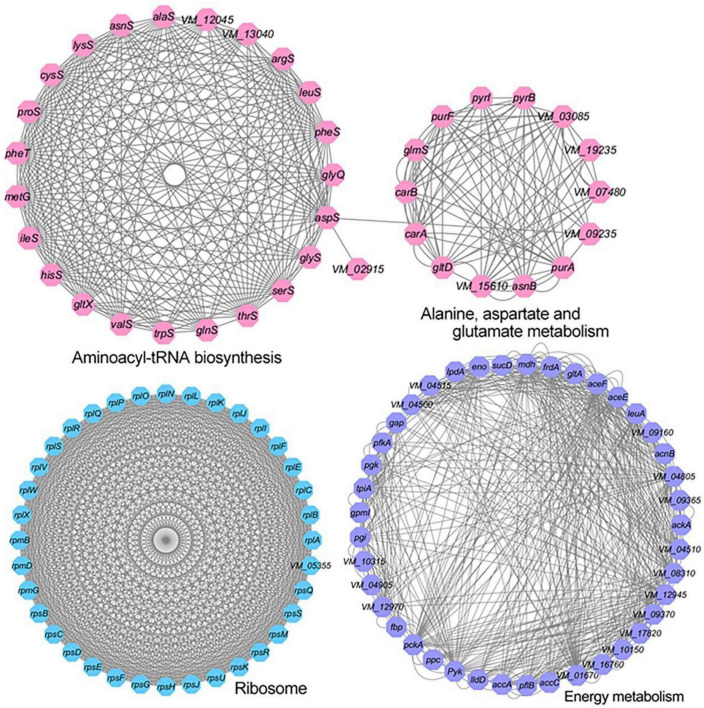
Protein–protein interaction network of aminoacyl tRNA biosynthesis, alanine, aspartate, and glutamate metabolism, and ribosome and energy metabolism related acetylated proteins in *V. mimicus*.

Protein–protein interaction plays a vital role in various biological processes. To further come to understand the metabolic pathways of acetylation regulation in *V. mimicus*, we used STRING software to analyze the PPI network for acetylated proteins. The results show that there are 571 network nodes (acetylated proteins) and 7,827 lines (interactions between proteins) constituting the network of the lysine acetylome. Previous studies reported that the interaction networks of acetylated proteins in *V. parahemolyticus* and *S. pneumoniae* were 502 network nodes and 8,627 direct physical interactions, and 392 network nodes and 4,995 direct physical interactions, respectively (Pan et al., 2014; Liu et al., 2018). Sixteen highly interconnected KEGG pathways of acetylated proteins were enriched in the global PPI network generated by STRING. The aminoacyl-tRNA biosynthesis, alanine, aspartate, and glutamate metabolism, ribosome, and energy metabolism (including TCA cycle, glycolysis/gluconeogenesis, and pyruvate metabolism) high-ranking interaction clusters are shown in Figure 4. The networks of ribosome (36 acetylated proteins) and aminoacyl-tRNA biosynthesis (24 acetylated proteins are mainly involved in protein translation) consist of 630 and 219 lines (direct physical interactions), respectively, showing that lysine acetylation modification plays an important regulatory role in these two pathways. The results were consistent with the acetylome of *V. parahemolyticus*, *V. alginolyticus*, and *S. pneumoniae* (Pan et al., 2014; Liu et al., 2018; Pang et al., 2019). PPI network analysis shows that acetylated proteins of *V. mimicus* comprise a dense protein interaction network, and offers a better way to further understand the functions of acetylated proteins in *V. mimicus*.

### Acetylated Proteins Involved in Bacterial Virulence

According to online VFDB software analysis, we found that 16 lysine acetylation sites spread in 13 proteins have a bearing on the virulence of *V. mimicus* (Table 1). Thirteen acetylated proteins have a hand in various pathogenic processes such as chemotaxis and motility, the secretion system, antiphagocytosis, and quorum sensing. Thus, we speculate that acetylation may play a role in bacterial virulence regulation. For chemotaxis and motility, including *cheR*, *cheV*, *cheY*, *VMB_14780*, *VMB_14810*, *VMB_16810*, and *VMB_28310*, functional studies demonstrate that chemotaxis and motility related genes play a pivotal role in the entry of bacteria into the host (Matilla and Tino, 2017). Proteins EpsE, ClpB, VgrG, and VMB_06040, associated with secretion systems, have been shown to play a major role in bacterial infection (Navarro-Garcia et al., 2019), especially that ClpB is mainly involved in regulating secretion of effector molecules related to type VI secretion systems and promotes survival of pathogenic bacteria (Alam et al., 2021); furthermore, its acetylation was also found in *V. alginolyticus* in our previous studies (Pang et al., 2019). Additionally, the gene encoding S-ribosylhomocysteine lyase (LuxS) is also found in *V. mimicus*; it might affect the pathogenesis by regulating QS (quorum sensing) (Sun et al., 2019; Cao et al., 2020). In addition, we also found that *luxO-luxR* coupled the main features of QS system in genus *Vibrio*, which were acetylated (Miyamoto et al., 2003). Thus, *V. mimicus* infection case has a strong relationship with a mixing effect of multiple virulence factors.

**TABLE 1:** Acetylated virulence factors in *V. mimicus.*

Protein	Gene name	Kac site	VF class
D2Y9X0	*cheR*	K35, K155	Chemotaxis and motility
D2YB90	*cheV*	K231	Chemotaxis and motility
D2YH36	*cheY*	K106	Chemotaxis and motility
D2YD81	*VMB_14780*	K42	Chemotaxis and motility
D2YD84	*VMB_14810*	K169	Chemotaxis and motility
D2YDT4	*VMB_16810*	K131	Chemotaxis and motility
D2YH34	*VMB_28310*	K211, K362	Chemotaxis and motility
D2YBG8	*epsE*	K190	EPS type II secretion system
D2YAQ4	*clpB*	K58	VAS type VI secretion system
D2YAR1	*vgrG*	K709	VAS effector proteins
D2YAQ7	*VMB_06040*	K346	VAS type VI secretion system
D2YDI6	*VMB_15830*	K187, K402	Antiphagocytosis
D2Y953	*LuxS*	K170	Quorum sensing

### Co-immunoprecipitation and Western Blotting Were Used to Verify LuxO and LuxR Acetylated Proteins

**FIGURE 5:**
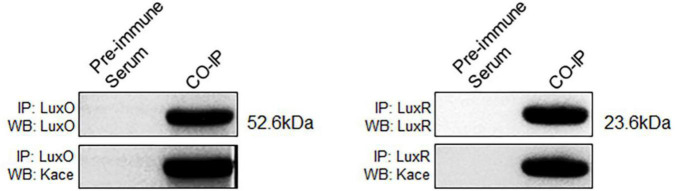
Validation of LuxO and LuxR by co-immunoprecipitation and Western blotting. LuxO and LuxR proteins were captured by specific antibodies (anti-LuxO and anti-LuxR), and validation by Western blotting with anti-LuxO and anti-LuxR (above), and anti-lysine acetylation antibodies (below).

LuxO and LuxR, two Kace proteins, were analyzed by co-immunoprecipitation (co-IP) and Western blotting to further prove the identified lysine-acetylated results in *V. mimicus*. The LuxO and LuxR proteins were enriched using anti-LuxO and anti-LuxR antibodies and visualized *via* Western blotting, which was performed with target proteins or anti-acetyl lysine antibody antibodies, respectively (Figure 5 and Supplementary Figure 2). The results state clearly that the acetylated modifications of LuxO and LuxR proteins were consistent with the proteomic data of lysine acetylation, which further verified our proteomic results.

## Conclusion

In summary, this study comprehensively analyzed the acetylome in *V. mimicus*, which provides a basis for further research on the regulation of lysine acetylation in the future. We identified 1,097 acetylation sites and 582 acetylated proteins, accounting for 15.5% of the total proteins in *V. mimicus*. In addition, the analysis of the conserved motifs proclaimed that the Kac is occupied by a leucine (L) residue at the + 1, −1, −2, or −7 position. Moreover, bioinformatics analyses indicate that lysine modification of proteins plays a central regulatory part in some biological processes, such as biosynthesis of secondary metabolites, biosynthesis of antibiotics, microbial metabolism in diverse environments, carbon metabolism, energy metabolisms, and virulence. In summary, this study totally reported the global protein acetylome in *V. mimicus* and provides a research basis for further study on the regulatory function of protein acetylation in *V mimicus*.

## Data Availability Statement

The datasets presented in this study can be found in online repositories. The names of the repository/repositories and accession number(s) can be found below: ProteomeXchange (accession: PXD028467).

## Author Contributions

HP and WL conceived the research project. JW, LY, and FZ performed the experiments. WL and NW performed the data analysis. HP, WL, RH, SM, and JJ interpreted the data and discussed the results. WL wrote the manuscript. All authors contributed to the article and approved the submitted version.

## Conflict of Interest

The authors declare that the research was conducted in the absence of any commercial or financial relationships that could be construed as a potential conflict of interest.

## Publisher’s Note

All claims expressed in this article are solely those of the authors and do not necessarily represent those of their affiliated organizations, or those of the publisher, the editors and the reviewers. Any product that may be evaluated in this article, or claim that may be made by its manufacturer, is not guaranteed or endorsed by the publisher.

## Footnotes

1
http://www.omicsbean.cn/

2
http://meme-suite.org/tools/momo?tdsourcetag=s_pcqq_aiomsg

3
https://string-db.org/

4
http://proteomecentral.proteomexchange.org

## Supplementary Material

Supplementary Table 1 Acetylated proteins and sites identified in *V. mimicus*.

Supplementary Table 2 The identified peptides in *V. mimicus*.
