# Supplementary material for: A Comprehensive Analysis of the Lysine Acetylome in the Aquatic Animals Pathogenic Bacterium Vibrio mimicus
Source: Front Microbiol. 2022 Feb 17;13:816968. doi: 10.3389/fmicb.2022.816968 (PMC8891801; doi:10.3389/fmicb.2022.816968)
Supplement: Supplementary file 3 [file Data_Sheet_1.docx]

**Supplement Figure 1**


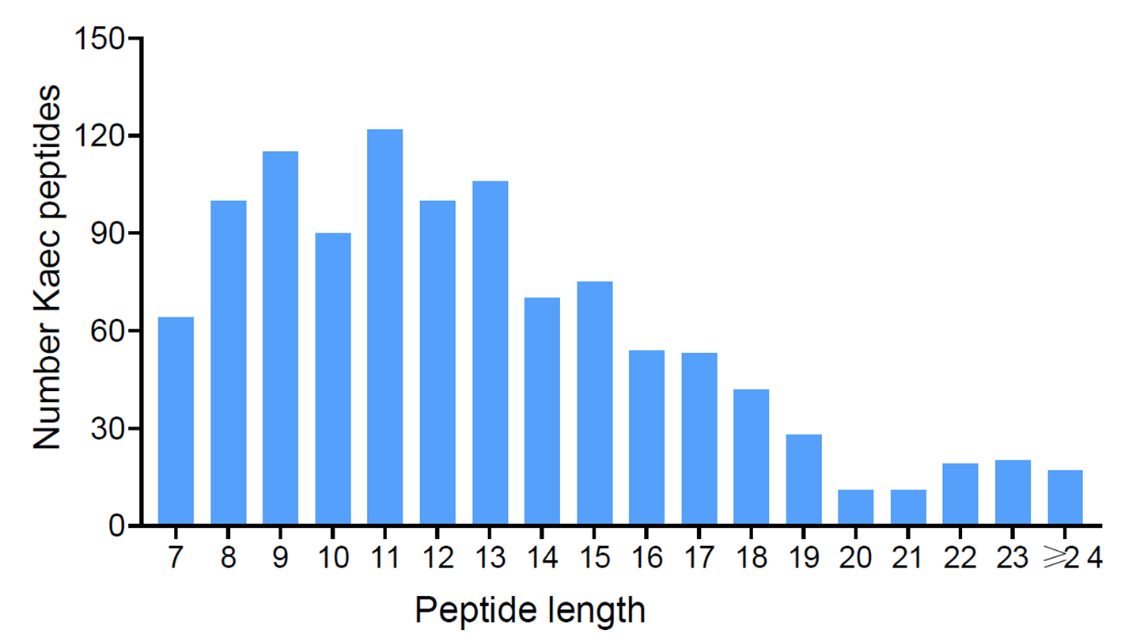


Supplement Figure 1 The distribution of acetylated peptides length.

**Supplement Figure 2**


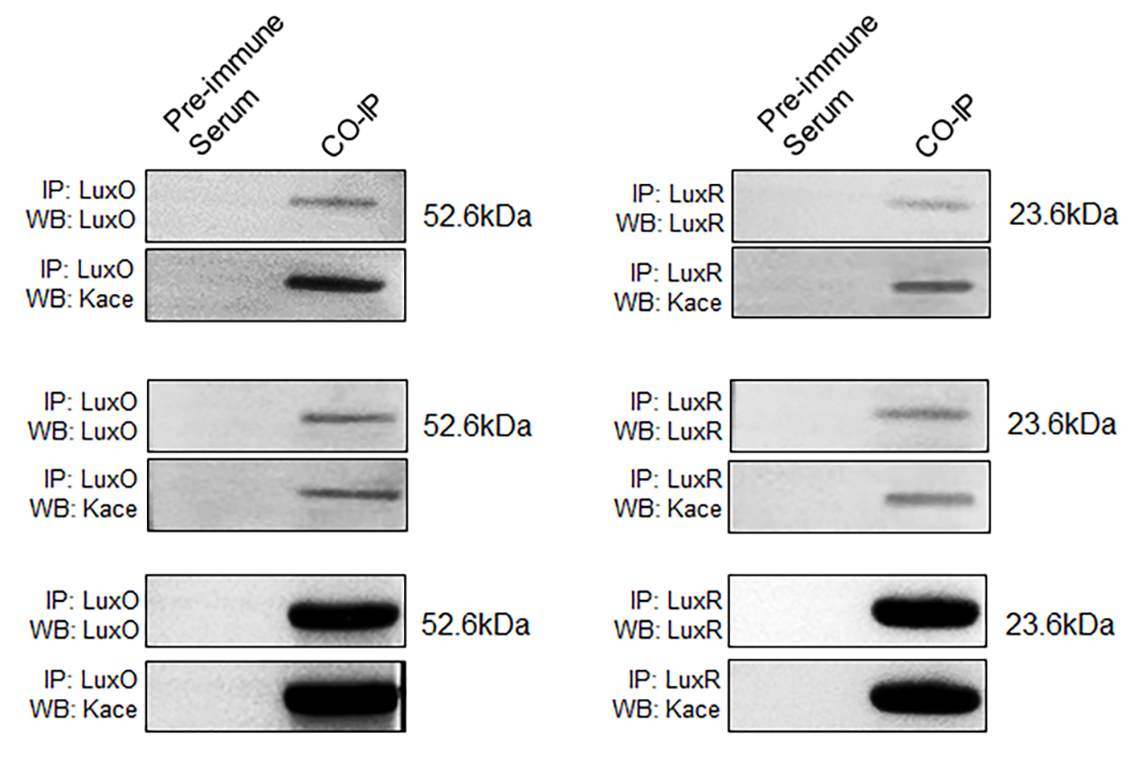


Supplement Figure 2 Validation of LuxO and LuxR by Co-Immunoprecipitation and Western blotting. Three repeats of Western blotting. The sample loading amount was low in the first two times and increased in the third time
